# Development of web-based dynamic nomogram to predict survival in patients with gastric cancer: a population-based study

**DOI:** 10.1038/s41598-022-08465-w

**Published:** 2022-03-17

**Authors:** Atefeh Talebi, Nasrin Borumandnia, Hassan Doosti, Somayeh Abbasi, Mohamad Amin Pourhoseingholi, Shahram Agah, Seidamir Pasha Tabaeian

**Affiliations:** 1 Colorectal Research Center, Iran University of Medical Sciences, Tehran, Iran; 2 Urology and Nephrology Research Center, Shahid Beheshti University of Medical Sciences, 1666663111 Tehran, Iran; 3 Department of Mathematics and Statistics, Macquarie University, Sydney, Australia; 4 Department of Mathematics, Isfahan (khorasgan) Branch, Islamic Azad University, Isfahan, Iran; 5 Gastroenterology and Liver Diseases Research Center, Research Institute for Gastroenterology and Liver Diseases, Shahid Beheshti University of Medical Sciences, Tehran, Iran; 6 Internal Medicine and Gastroenterology, Colorectal Research Center, Iran University of Medical Sciences, Tehran, Iran; 7 Gastroenterology and Hepatology, Iran University of Medical Sciences, Tehran, Iran

**Keywords:** Cancer, Gastroenterology

## Abstract

Gastric cancer (GC) is the fifth most frequent malignancy worldwide and the third leading cause of cancer-associated mortality. The study’s goal was to construct a predictive model and nomograms to predict the survival of GC patients. This historical cohort study assessed 733 patients who underwent treatments for GC. The univariate and multivariable Cox proportional hazard (CPH) survival analyses were applied to identify the factors related to overall survival (OS). A dynamic nomogram was developed as a graphical representation of the CPH regression model. The internal validation of the nomogram was evaluated by Harrell’s concordance index (C-index) and time-dependent AUC. The results of the multivariable Cox model revealed that the age of patients, body mass index (BMI), grade of tumor, and depth of tumor elevate the mortality hazard of gastric cancer patients (P < 0.05). The built nomogram had a discriminatory performance, with a C-index of 0.64 (CI 0.61, 0.67). We constructed and validated an original predictive nomogram for OS in patients with GC. Furthermore, nomograms may help predict the individual risk of OS in patients treated for GC.

## Introduction

Gastric cancer (GC) is one of the most common malignancies, with high incidence and mortality rates, and ranks as the fifth most frequent cancer and the third leading cause of cancer-related deaths globally^1^. The cancer is most commonly diagnosed in men among five countries (Bhutan, China, Kyrgyzstan, Mongolia, and Vietnam)^2^. Despite the fact that the incidence of GC has reduced during the past decades in European countries, the prognosis stays poor^3^. Also, the 5-year overall survival rate for GC patients is approximately 25 percent in the West^4^. Though the incidence of GC has decreased in Western countries in recent decades, its incidence has remained high in some Eastern countries (e.g., China, Japan, and Korea). Basis on the cancer registry of Turkey, GC may be the 5th most common malignancy in both men and women^5^. countries, two-thirds of new cases of stomach cancer are recorded, and survival is still low in both developed and developing countries^6^.

The incidence is approximately 7300 cases per year among the Iranian population, which is the most common cancer in men^7^. Moreover, mortality from GC can be the first leading factor of death due to cancer in both sexes^8^. However, the incidence of the disease was higher in men than in women^9^. Considering the low rate of 5-year survival of GC patients, identification and control of predictive factors remain the essential prevention methods^10^.

Notwithstanding these technologies and therapeutic strategies have progressed over the last several decades, but GC patients’ survival and risk factors remain unsatisfactory. It is crucial to recognize prognostic factors for those patients.

According to various statistical analyses, many researches have been performed to evaluate the prognosis factor on the survival of patients with GC^10,11^. Those methods incorporate several variables to predict a particular endpoint using traditional statistical methods (e.g., logistic or CPH regression models)^12,13^. Other studies have surveyed machine learning methods, which are a branch of artificial intelligence (AI) and computer science, on GC data^14–17^. Almazat et al. applied the Kaplan–Meier method to estimate overall survival in patients with GC^18^. The Cox and Frailty models of survival analysis were used and then compared with C-index^13^. Other researchers have presented nomograms, which are the graphical representation to intuitive perception of clinicians in Colorectal cancer (CRC) and GC patients^11,19,20^. A nomogram, a simple graphical visualization combining and quantifying all independent prognostic factors, plays an increasingly vital role in medical sciences and clinical studies^21^. Moreover, nomograms play a significant role in improving prognostic accuracy by combining all independent prognostic factors and quantifying their risks^22,23^. Nomogram, can be considered as a predictive statistical model for individual patients, reveals benefits than the conventional staging systems in predicting the outcomes of long-term survival in patients^24^. To the best of our knowledge, this study was the first survey on the predictive model regarding to nomogram presentation and OS for GC in Iran.

The objective of this study was to establish a CPH regression model to explore prognostic factors for OS in patients who underwent D2 radical gastric cancer surgery. Then, a more dynamic nomogram was constructed to predict overall survival based on a relatively large historical cohort of patients with GC.

## Materials and methods

### Patients and data sources

The demographical and clinicopathological characteristics of 733 GC patients were extracted from a tertiary University-Hospital of Iran, Taleghani Hospital in Tehran, between 2013 and 2020. This research complies with the principles of the Helsinki Declaration. We obtained the patients’ informed consent to be allowed to use their medical information. The methods were carried out in accordance with the relevant guidelines and regulations. The Ethics Committee of Iran University of Medical Sciences approved the study (Ethical code: IR.IUMS.REC.1399.122).

### Demographic and clinical variables

Survival time, based on months elapsed from the cancer diagnosis until death, was considered the outcome variable. The demographical and clinical variables, including sex, marital status, smoking status, body mass index (BMI), family history, type of treatment, grade of tumour, depth of tumour, number of involved lymph nodes were predictors. The patients’ survival status was collected based on alive or dead.

### Statistical analysis

The continuous variables were described as mean ± SD. Also, the frequency and percentage of categorical variables were reported. Missing data were imputed by fully conditional specification^25^. The Kaplan–Meier was used in order to estimate the survival function. We applied a univariable CPH model to explore the relationship between a patient’s survival and explanatory variables. The selected variables with P < 0.2 in the univariable analysis were subjected to multivariable regression modelling. Then, the nomogram was illustrated according to the multivariable CPH model. At last, C-index, as a global index for validating the predictive ability of a survival model, and time-dependent area under the roc curve were calculated to assess the internal validation. Also, internal calibration using bootstrap resampling was assessed by plotting the predicted probabilities from the model versus actual survival probabilities. The analysis was perform using the SPSS 23 and Stata 11. The survival, DynNom, rms, and hdnom packages in R 4.1.0 software were used to create a dynamic nomogram and to perform validation and calibration. Additionally, decision curve analysis (DCA) was applied with the function of “dcurves”. When the net benefit of a model is greater than curing in both all and none group of patients, the model can be considered as a clinical utility. The decision curve model can be compared with serious cases that curing all patients or none. If a model has acceptable level of advantage in a wide range of clinically reasonable preferences, the model or test can be advised.

## Results

The study population consisted of 733 confirmed patients with GC who underwent treatment. The median of follow-up time is 9.55 months (IQR = 4–19.13, range 0.1–84). The mean ± SD age of patients was 59.49 ± 13.47 years, with ranges from 14 to 89 years. 932 (69.1%) of patients were male, and 417 (30.9%) were female. Six hundred ninety-nine (51.8%) patients were censored, and 643 (47.7%) of patients died at the end of follow-up. Other demographic and pathological characteristics of GC patients are given in Table 1. Also, the table revealed factors related to survival rate according to the univariable CPH regression model.

**Table 1:** Univariable Cox regression analyses for survival in patients with gastric cancer.

	Status		β	HR(CI)	P value
	Censored	Dead			
Survival time (month), median (IQR)	10.8 (2.9–21.8)	10.0 (5.3–16.7)		–	–
Age, mean (SD)	61.0 (12.45)	60.3 (13.26)	0.01	1.01 (1.005, 1.02)	0.003
**Sex**					
Male	318 (60.9)	204 (39.1)	0.02	1.02 (0.79, 1.32)	0.877
Female	132 (62.6)	79 (37.4)	Ref*	Ref	
**Marital status**					
Married	441 (61.3)	278 (38.7)	0.30	1.35 (0.37, 4.89)	0.543
Single	8 (80.8)	2 (20.0)	Ref	Ref	
**Smoking**					
Never used	285 (63.6)	165 (36.7)	Ref	Ref	0.272
Previous or current user	143 (57.0)	108 (43.0)	0.13	1.14 (0.90, 1.45)	
**BMI**					
≤ 18.5	49 (46.7)	56 (53.3)	0.28	1.33 (0.96, 1.84)	0.082
18.5–25	192 (63.6)	110 (36.4)	Ref	Ref	
25–30	48 (80.0)	12 (20.0)	−0.48	0.61 (0.34, 1.12)	0.114
≥ 30	8 (72.7)	3 (27.3)	−0.46	0.62 (0.19, 1.97)	0.426
**Family history**					
No	314 (61.0)	201 (39.0)	Ref	Ref	0.152
Yes	108 (62.1)	66 (37.9)	−0.19	0.82 (0.62, 1.07)	
**Type of treatment**					
Surgery treatment	288 (64.4)	159 (35.6)	Ref	Ref	
Other treatments	162 (56.6)	124 (43.4)	0.47	1.61 (1.27, 2.04)	< 0.001
**Tumour grade**					
Well	76 (68.5)	35 (31.5)	Ref	Ref	
Moderately	89 (65.0)	48 (35.0)	0.17	1.19 (0.77, 1.85)	0.423
Poorly	120 (60.0)	80 (40.0)	0.36	1.44 (0.97, 2.14)	0.072
Undifferentiated	165 (57.9)	120 (42.1)	0.36	1.43 (0.98, 2.09)	0.061
**Depth of tumour**					
T1	13 (81.3)	3 (18.8)	−0.98	0.37 (0.12, 1.12)	0.078
T2	55 (80.9)	13 (19.1)	−1.08	0.33 (0.19, 0.57)	< 0.001
T3	175 (68.1)	82 (31.9)	−0.46	0.62 (0.48, 0.81)	< 0.001
T4	90 (41.9)	125 (58.1)	Ref	Ref	
**Number of involved lymph node**					
N1	86 (66.7)	43 (33.3)	Ref	Ref	
N2	159 (62.4)	96 (37.6)	0.10	1.11 (0.74, 1.66)	0.582
N3	44 (68.8)	20 (31.3)	0.02	1.02 (0.63, 1.66)	0.914

*Ref: Reference.

The results showed that age of diagnosing, BMI, family history, type of treatment, grade of tumour, and depth of tumour were significant in the univariable CPH model (P < 0.05).

**Figure 1:**
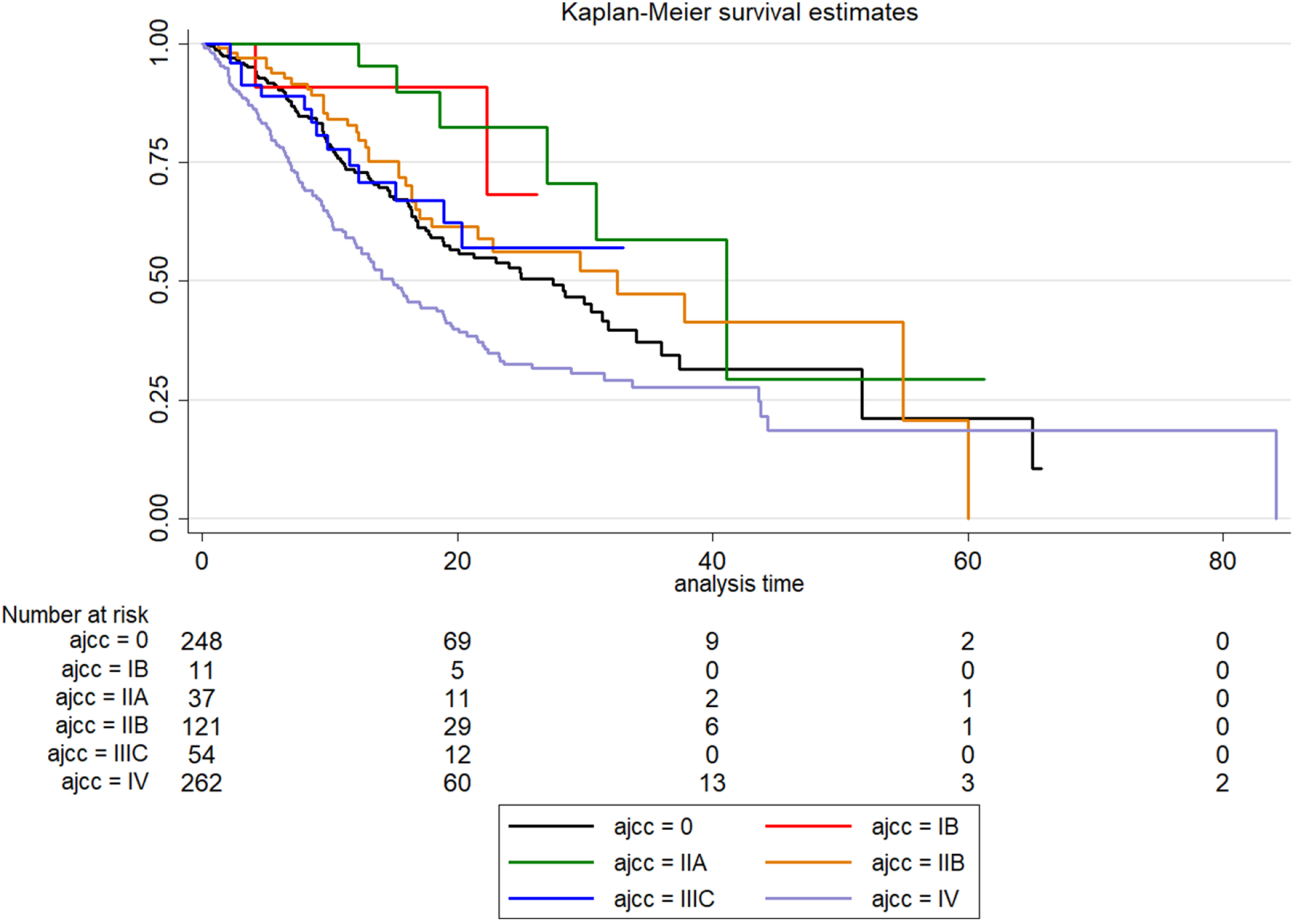
Kaplan–Meier survival curves by American joint committee on cancer staging, along with the number at risks in gastric cancer patients.

Figure 1 revealed the Kaplan–meier according to American Joint Committee on Cancer (AJCC) staging, which the number at risks were listed below.

The results of the multivariable CPH were presented in Table 2. Variables that had P < 0.2 in the univariable analysis were candidates for the multivariable regression analysis. The table showed that age of diagnosis, BMI, grade of the tumour, and depth of tumour are significant in the multivariable CPH model (P < 0.05).

**Table 2:** Multivariable Cox regression model for survival in GC patients.

	β	HR (CI)	P value
Age	0.01	1.01 (1.005, 1.02)	0.003
**BMI**			
≤ 18.5	0.24	1.27 (0.97, 1.66)	0.075
18.5–25	Ref	Ref	
25–30	−0.61	0.54 (0.32, 0.90)	0.020
≥ 30	0.18	1.20 (0.69, 2.08)	0.518
**Family history**			
No	Ref	Ref	
Yes	−0.07	0.93 (0.70, 1.23)	0.629
**Type of treatment**			
Surgery treatment	Ref	Ref	
Other treatments	0.28	1.33 (0.99, 1.78)	0.057
**Tumour grade**			
Well	0.03	1.04 (0.69, 1.56)	0.853
Moderately	0.20	1.22 (0.84, 1.76)	0.281
Poorly	0.45	1.57 (1.14, 2.15)	0.005
Undifferentiated	Ref	Ref	
**Depth of tumour**			
T1	−0.96	0.38 (0.15, 0.95)	0.039
T2	−0.94	0.39 (0.22, 0.68)	0.001
T3	−0.40	0.66 (0.49, 0.90)	0.009
T4	Ref	Ref	

The result showed that for every 10 years of increasing the age, the hazard rate increases by 10% (HR = 1.01, P value < 0.05). The HR in patients with the overweight range was 46% less the than normal group (HR = 54%, P < 0.05); however, obese patients had higher HR than normal weight, which is non-significant (HR = 1.2, P = 0.518).

Also, the hazard ratio in patients who underwent chemotherapy, radiotherapy, and immunotherapy, presented as other treatments in the table, is 33% more than the people who had surgery; however, the type of treatment was non-significant (HR = 1.33, P = 0.057). Moreover, HR of tumour grade in patients with undifferentiated tumour grade was 57% more than people with well grade (HR = 1.57, P < 0.05). When the depth of the tumour deteriorated, the HR was soared significantly in GC patients. Thus, the higher the tumour depth, the higher HR (P < 0.05).

**Figure 2:**
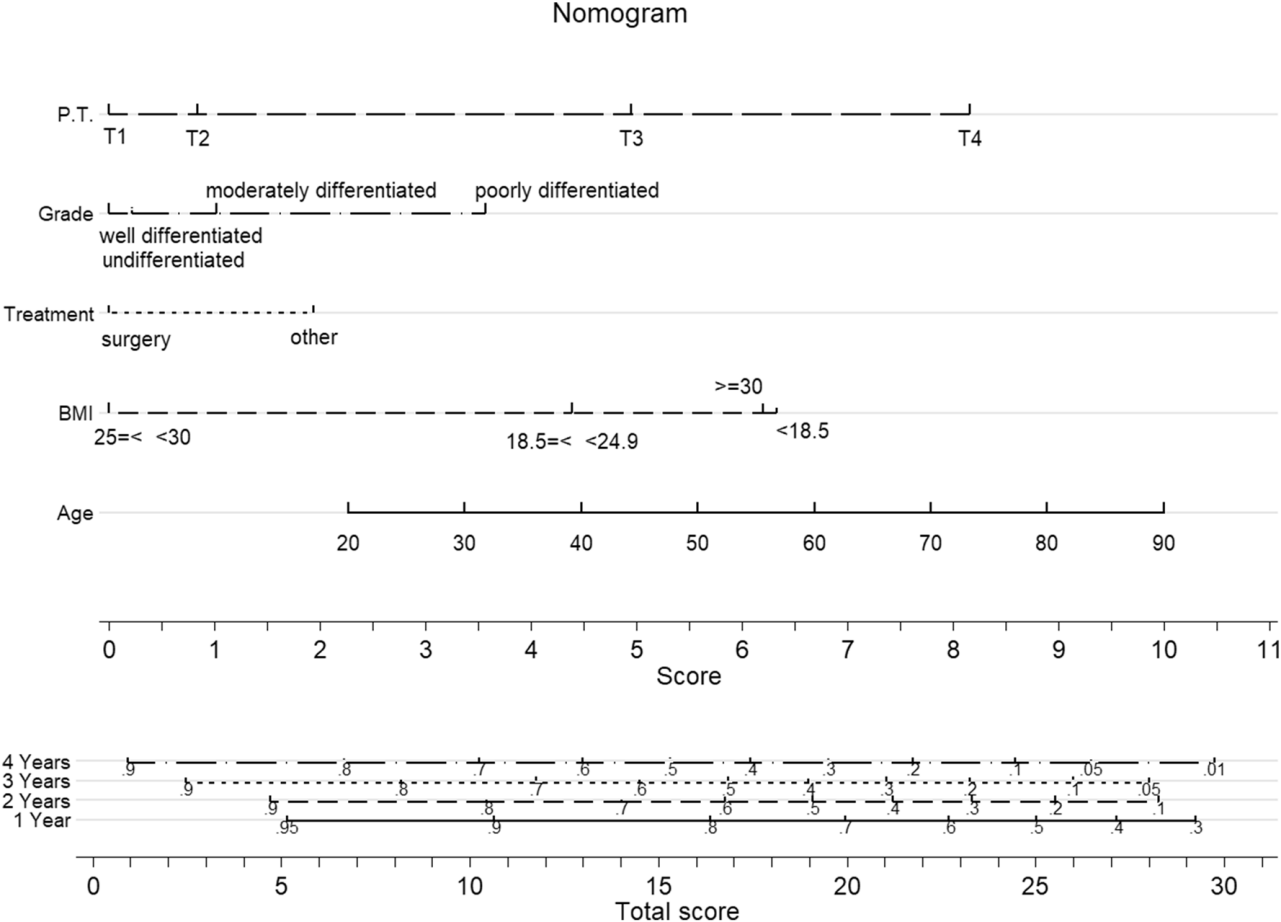
Predictive nomogram for survival in Gastric cancer patients.

**Figure 3:**
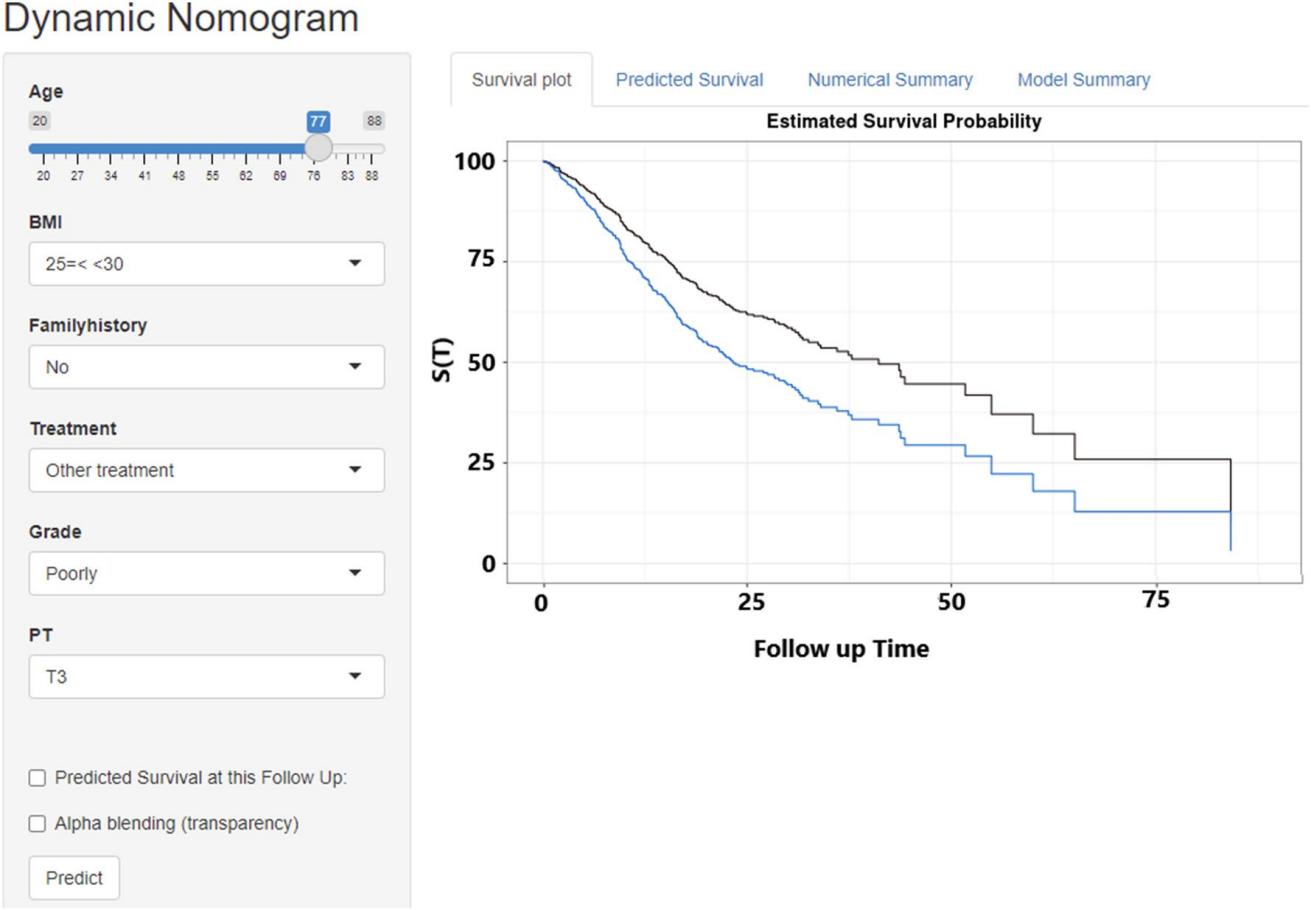
Web-based survival rate calculator (Dynamic Nomogram (shinyapps.io)) to predict the survival of GC patients; Two patients who are 77 years old, no surgery, no family history, PT3 and 25 ≤ BMI < 30, but have different grade, well (black line) and poorly (blue line), according to the web survival rate calculator.

The results of the multivariable CPH model were presented as a nomogram in Fig. 2. The probability of survival for a GC patient can be predicted at a specific time point using this nomogram. The patient’s characteristics have been plotted on each variable axis. To predict the survival probability of a patient, a vertical line is drawn from the patient’s characteristics value to the top points scale. In this way, the number of points that were assigned by that variable value is determined. Then, the points from each variable value are summed. Finally, the sum on the total points is vertically projected onto the bottom axis, and a personalized probability survival time is obtained. Figure 3 shows the image of a web-based nomogram which is accessible in the https://nbshiny.shinyapps.io/GastricDynNom/. This is very simple-to-use web-based nomogram for convenient application, which can aid personalized treatment and clinical decision-making. This dynamic nomogram considers the sliders for covariates variable, bounded on the observed ranges, and drop-down boxes for categorical ones.

### Internal validation and calibration

**Figure 4:**
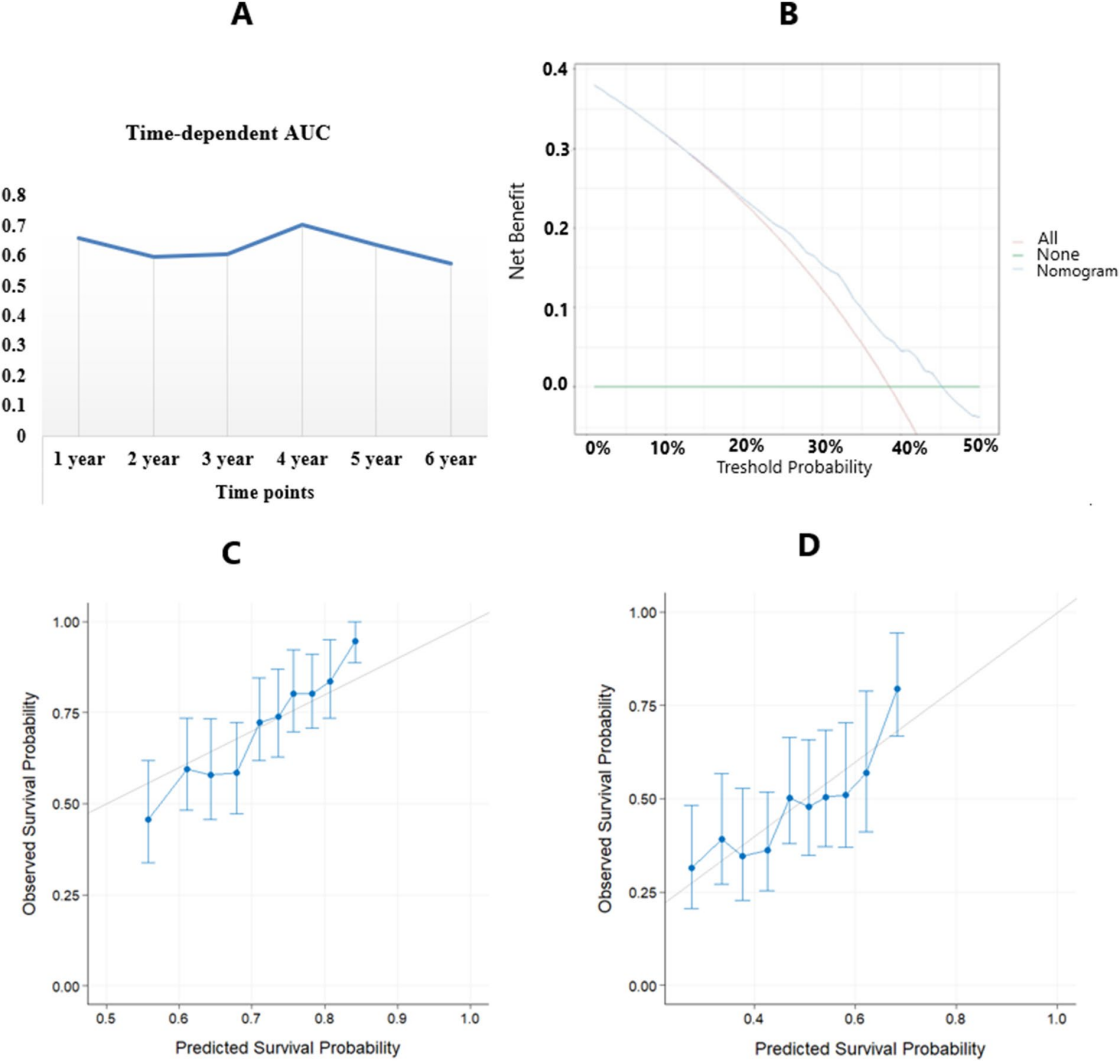
(**A**) Time-dependent AUC summary at evaluation time points. (**B**) Decision curve analysis. (**C**) Internal calibration using bootstrap resampling at 1 year and 2 years.

The internal validation was checked using C-Index and time-dependent AUC at evaluation time points. The C-index was calculated as 0.64 (CI 0.61, 0.67) also, we validate the performance of the CPH model with bootstrap resampling every year from the first year to the sixth year. In addition to, the C-index of the presented model, 0.64, was slightly less that of the AJCC clinical staging 0.68. The time-dependent AUC at 1, 2-, 3-, 4-, 5- and 6-years follow-up have been presented in Fig. 4A. Based on DCA, if the threshold probability be > 0.45, the developed nomogram is superior in predicting survival in all of the patients (Fig. 4B). In addition, the internal calibration using bootstrap resampling was assessed by plotting the predicted probabilities from the model versus actual survival probabilities. In this way, the samples were split into ten risk groups, and the survival probabilities at 1 and 2 years were obtained and summarized as calibration plots in Fig. 4C.

## Discussion

This study provided a significant contribution through the use of a historical cohort of patients with GC who were treated in Iran from 2009 to 2020. As far as is known, this is the first study of nomogram in GC patients of Iranian population, known as a user-friendly clinical tool with an acceptable sample size and long-term follow up. In our study, we applied a web-based nomogram that can be used to predict the survival probability. The multivariable CPH model presented that age of diagnosing, BMI, family history, type of treatment, grade of tumour, and depth of tumour were statistically significant. Furthermore, we construct a nomogram to predict OS, which could provide individualized estimates of potential survival and aid individualized management decisions for GC. The C-index, calculated as 0.64 (CI 0.61, 0.67), was applied to evaluate the model internal validation, and found that the prognostic model has high accuracy. The C-index of 0.68 in AJCC clinical staging surpass the C-index of the presented model. Moreover, the time-dependent AUC was obtained to validate the performance of model, which was more than 60% for 1-, 3-, and 5-year of survival.

Nomogram is a precise and useful clinical tool that can help clinicians predict the probability of an outcome event, that is, survival time. A variety of nomograms have been built to predict the therapeutic benefits, the postoperative survival rate in patients with GC^26–28^. Mu et al. predicted the long-term survival of 421 GC patients, who underwent D2 radical lymphadenectomy, using survival model and establish a nomogram^27^. They calculated C-index of the model that was 0.76 for internal verification. Their significant factors were tumour staging, location of tumour, BMI, neural and vessel invasion. In our investigation, the age, grade of tumour and depth of tumour were considered as the main factor in multivariable CPH; also, the C-index was calculated 0.64. A study was done by Han et al. to predict survival after D2 gastrectomy for GC patients^29^. The C-index for OS was 0.69, and also, they established a nomogram predicting 5- and 10-year overall survival after D2 gastrectomy for gastric cancer. Also, another gastric cancer study multivariable Fine and Gray regression model to predict disease-specific mortality (DSM) that considered competing risks^30^. The goal of the study was to progress the first pre-treatment gastric cancer nomogram for predicting DSM that represented a acceptable discrimination in the new nomogram. Their result showed that the newly advanced nomogram perfectly predicted DSM, which can be used for patient advising in medical practice. In this study, their C-index of the model was 0.887 as well as the AJCC clinical staging 0.794. However, in our study the C-index of our model, equal to 0.64, was slightly less that of the AJCC clinical staging 0.68.

Here, we constructed a nomogram to predict the survival rate in GC patients. According to previous studies, a C-index > 0.6 indicated that the built model had an acceptance accuracy^29,31,32^. The value of this index was consistent with our study. In general, a few studies have applied AUC to predict the OS^26,28,33^. The AUC values of ROC were more than 60% for 1-, 3-, and 5-year of survival, which are compatible with our study. In addition, DCA was drawn to evaluate the clinical application value of the nomogram^28,30–32,34^. Lu et al. used a nomogram to predict recurrence-free survival and the advantages of adjuvant chemotherapy after radical resection in high stage GC patients^31^. They applied CPH model to identify predictive factors for RFS; moreover, established a novel nomogram for GC after radical resection.

Our multivariable CPH regression model discovered that age of diagnosis, BMI, grade of the tumour and tumour depth were independent risk factors in GC. Most of the previous studies focused on independent variables associated with GC and found that tumour depth, differentiation grade, size, and lymphatic invasion were closely associated with patients’ survival^35,36^. Lu et al. reported age, differentiation, tumour size, number of examined lymph nodes, pT stage, pN stage, and adjuvant chemotherapy as associated with GC^31^. They showed that the hazard ratio increased with age, which means that, the older patients get, the higher the hazard. Similarly, our study found that age and pT stage were significant in the CPH model. In our multivariable CPH regression model, the groups with 25 ≤ BMI < 30 had significantly higher than the group with a BMI ≤ 18.5 cm, suggesting that the BMI was a powerful predictor in patients with GC. The built nomogram finally corroborated that the BMI was one of the main risk factors in predicting survival of GC patients^27,32^. Regarding the grade of tumour, most previous studies reported that the tumour grade was a main predictor for patients with GC^14,15,22,26,27,29,31^. Similarly, our study found the grade of tumour can be a strong predictor in patients with GC. That means the worse the tumor grade, the greater the hazard ratio.

### Study limitation

The key strength of this study is the long-term follow-up period. The second strength of the study is to use web-based nomogram that any expert can calculate the overall survival probability. Also, we had several limitations. First, some variables, such as Helicobacter pylori infection status, location of tumour, demarcation line of tumour lesion, tumour markers, nutritional status, and Charlson Comorbidity Index, may also be potential risk factors in patients with GC and need to be incorporated into our model. Second, statistical analysis was performed using internal validation. It is suggested that in future studies, external validation can be performed using another test dataset. Third, the key limitation of the present survey was the small number of sample size in a center that recommend to larger sample size in similar studies. Forth limitation is to collect some variables, such as BMI and age in the form of continuous instead of categorical variable^37^.

## Conclusion

We successfully established a novel nomogram using patient data from the GC database in Taleghani University-Hospital. Furthermore, the age at diagnosing, BMI, tumour grade, depth of tumour made a significant contribution in predicting OS of patients with GC.

## Abbreviations

AI: Artificial intelligence
AJCC: American Joint Committee on Cancer
AUC: Area under the curve
BMI: Body mass index
C-index: Concordance index
CI: Confidence interval
CRC: Colorectal cancer
CPH: Cox proportional hazard
DCA: Decision curve analysis
DSM: Disease-specific mortality
GC: Gastric cancer
HR: Hazard ratio
IQR: Interquartile range
OS: Overall survival
RFS: Recurrence-free survival
ROC: Receiver operating characteristic
SD: Standard deviation
